# A Wearable System for Attenuating Essential Tremor Based on Peripheral Nerve Stimulation

**DOI:** 10.1109/JTEHM.2020.2985058

**Published:** 2020-04-06

**Authors:** Jeonghee Kim, Thomas Wichmann, Omer T. Inan, Stephen P. Deweerth

**Affiliations:** 1 Quantitative Neuro Rehabilitation LaboratoryDepartment of Engineering Technology and Industrial DistributionTexas A&M UniversityCollege StationTX77843USA; 2 Department of NeurologySchool of MedicineEmory University 1371 AtlantaGA30322USA; 3 School of Electrical and Computer EngineeringGeorgia Institute of Technology 1372 AtlantaGA30332USA; 4 Coulter Department of Biomedical EngineeringGeorgia Institute of Technology 1372 AtlantaGA30332USA; 5 P.C. Rossin College of Engineering and Applied ScienceLehigh University 1687 BethlehemPA18015USA

**Keywords:** Essential tremor, kinetic tremor, tremor measurement, tremor modulation, wearable peripheral nerve electrical stimulation

## Abstract

Objective: Currently available treatments for kinetic tremor can cause intolerable side effects or be highly invasive and expensive. Even though several studies have shown the positive effects of external feedback (i.e., electrical stimulation) for suppressing tremor, such approaches have not been fully integrated into wearable real-time feedback systems. Method: We have developed a wireless wearable stimulation system that analyzes upper limb tremor using a three-axis accelerometer and that modulates/attenuates tremor using peripheral-nerve electrical stimulation with adjustable stimulation parameters and a real-time tremor detection algorithm. We outfitted nine subjects with tremor with a wearable system and a set of surface electrodes placed on the skin overlying the radial nerve and tested the effects of stimulation with nine combinations of parameters for open- and closed-loop stimulation on tremor. To quantify the effects of the stimulation, we measured tremor movements, and analyzed the dominant tremor frequency and tremor power. Results:Baseline tremor power gradually decreased over the course of 18 stimulation trials. During the last trial, compared with the control trial, the reduction rate of tremor power was 42.17 ± 3.09%. The dominant tremor frequency could be modulated more efficiently by phase-locked closed-loop stimulation. The tremor power was equally reduced by open- and closed-loop stimulation. Conclusion: Peripheral nerve stimulation significantly affects tremor, and stimulation parameters need to be optimized to modulate tremor metrics. Clinical Impact: This preliminary study lays the foundation for future studies that will evaluate the efficacy of the proposed closed-loop peripheral nerve stimulation method in a larger group of patients with kinetic tremor.

## Graphical abstract

This study proposes and quantitatively assesses the feasibility of a real-time wearable tremor modulation system that provides constant-voltage peripheral-nerve stimulation. The results of our analysis show that our system significantly reduces kinetic tremor frequency and power, demonstrating the potential usefulness of peripheral-nerve stimulation as a treatment for essential tremor.

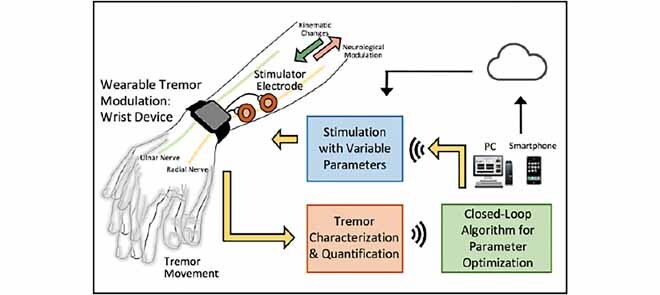

## I. Introduction

Tremor, an abnormal oscillatory movement, can be observed in patients with neurological disorders such as essential tremor (ET) and Parkinson’s disease (PD) [1]. Tremor can be characterized as resting tremor, kinetic tremor, or postural tremor [2], [3]. Tremor associated with PD usually occurs at rest. In contrast, tremor associated with ET occurs with movement. ET can be very debilitating, interfering with movements that require a high degree of dexterity and precision. Tremor frequency, typically ranging between 4 and 12 Hz, varies among patients and among tremor etiologies [1]–[4]. More than 90 percent of ET patients experience arm tremor [4].

Oral medications such as primidone or beta receptor blockers are commonly used to ameliorate kinetic or postural tremors [5]. These medications are beneficial in approximately 60 percent of patients who use them. The most common reason for failure of these pharmacologic strategies is lack of effectiveness and/or the emergence of significant side effects (e.g., fatigue, nausea, dizziness, ataxia, sedation) [5]. Repeated muscle injections of botulinum toxin [6]–[8] are also a therapeutic option. Deep-brain stimulation (DBS) of the thalamic ventral intermediate (VIM) nucleus is frequently used to treat medication-resistant cases of ET. While highly effective [9]–[15], the use of DBS is costly, and requires an invasive brain surgery.

Researchers have also explored how external stimulation could affect tremor [16]–[25]. Several studies explored the effects of open-loop stimulation of flexor and extensor muscles involved in tremor, finding that the stimulation could significantly attenuate tremor [16]–[18]. Other studies applied cutaneous/peripheral nerve stimulation to the hand or wrist to suppress tremor. The authors of these studies hypothesized that cutaneous/peripheral sensory afferent inputs to the spinal cord or the brain could modulate tremor [19]–[24]. None of these studies, however, used a fully integrated wearable device or closed-loop approaches.

**FIGURE 1.**
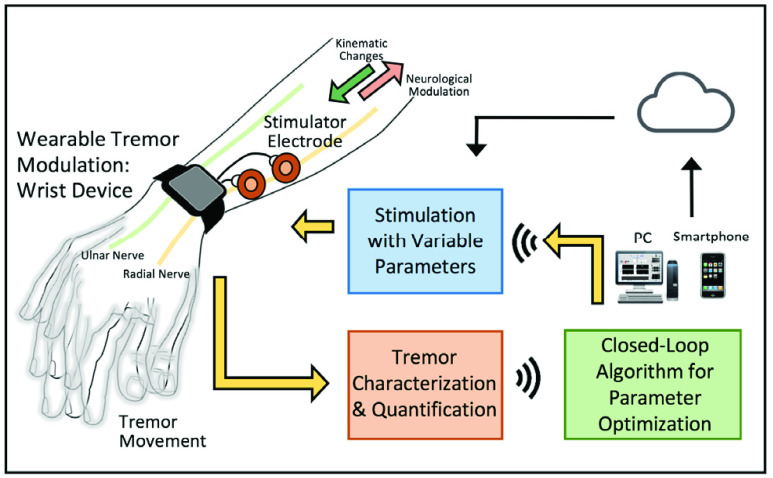
Overview of the wearable tremor-modulation system used in this study. Using a three-axis accelerometer, the system quantifies tremor characteristics (frequency and severity) and activates peripheral-nerve electrical stimulation to modulate/attenuate tremor movements. Off-board signal processing detects the current tremor status, applies stimulation based on the current tremor status, and ultimately maximizes the efficacy of tremor suppression by providing real-time stimulation parameter optimization (future work).

Since the tremor movements of ET are associated with certain postures and/or activities, our purpose is to explore the contribution of sensory modulation to the tremor movements of ET. We hypothesize that sensory signals (i.e., tactile and proprioceptive) contribute to the development of tremor and are closely related to its onset. Although the anatomical structures and pathways involved in generating ET [26], [27] and the effects of external stimulation of peripheral nerves within these pathways are not well defined [19]–[24], we also hypothesize that the stimulation of peripheral nerves within a proper range of the parameters modulates the characteristics of tremor and that this approach can reduce tremor. In this study, we explored how electrical stimulation applied to peripheral nerves with a variety of settings (e.g., timing, frequency, duration) changes the characteristics of tremor. To accomplish this goal, we developed a wearable tremor modulation system that uses peripheral nerve stimulation and real-time parameter updates (Fig. 1) and evaluated the feasibility of changing tremor with this system at various stimulation parameters.

## II. Methods

We developed the necessary hardware and software for a wireless wearable system that applies electrical stimulation to peripheral nerves, with real-time adjustments of stimulation parameters based on data from a built-in three-axis accelerometer. To evaluate the effect of the stimulation, we collected data from 18 stimulation combinations of stimulation parameter sets with and without stimulation while the subjects showed tremor, elicited by performance of a “bean transfer” task. Accelerometer data were used to analyze two objective tremor metrics. In addition, we used subjective and qualitative tremor assessments (tremor rating scores and questionnaires).

### A. Wearable Wrist Device

**FIGURE 2.**
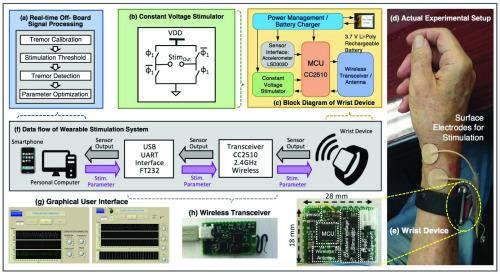
Details of the wearable tremor-modulation system. The system consists of a wearable wrist device that contains an accelerometer, an electric stimulation unit, a 2.4 GHz transceiver, and an off-board signal processing system linked to the wearable device. (a) Off-board signal processing flow for tremor detection and parameter optimization, (b) the voltage mode stimulator (biphasic stimuli are generated by alternating between turning on the electronic switches $\Phi _{1}$ and $\overline {\Phi }_{1}$, (c) the wearable wrist device, (d) experimental apparatus, including the wrist device and a pair of stimulating surface electrodes, (e) custom-designed printed circuit boards for the wrist device, (f) overall data flow from a PC or smartphone to the wrist device, (g) graphical user interface for the experimental setup (i.e., stimulation threshold setting and a phase-locked stimulation trial), and (h) a custom-designed wireless transceiver for the wrist device.

**FIGURE 3.**
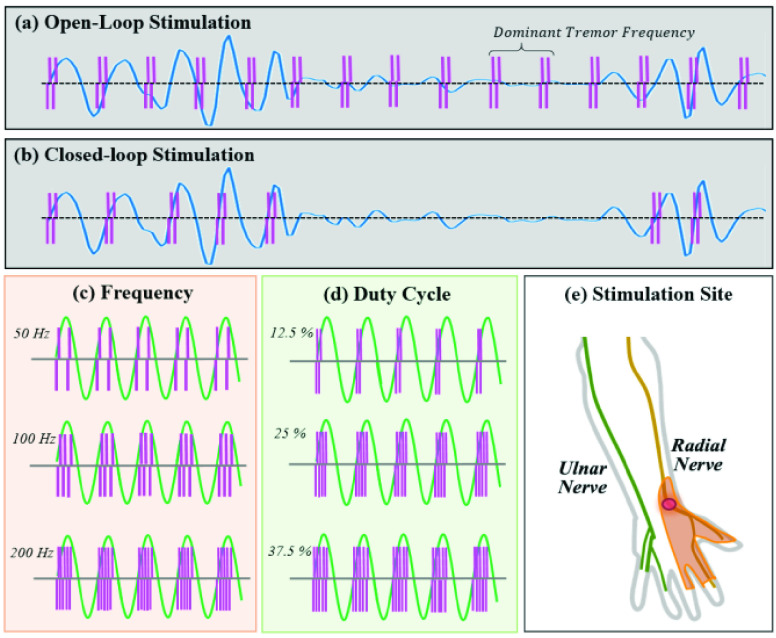
Stimulation parameters in this study: Example stimulation onsets for (a) open-loop and (b) stimulation phase-locked (at $0\pi $ of the tremor cycle) to the tremor movement. The nine combinations of stimulation parameter sets consisted of (c) three stimulation frequencies (Freq. = 50, 100, and 200 Hz), and (d) three duty cycles (Duty = 12.5. 25, and 37.5% of a tremor cycle). All combinations of stimulations were applied to (e) the site of the radial nerve and the skin territory affected by radial nerve stimulation (shaded).

**FIGURE 4.**
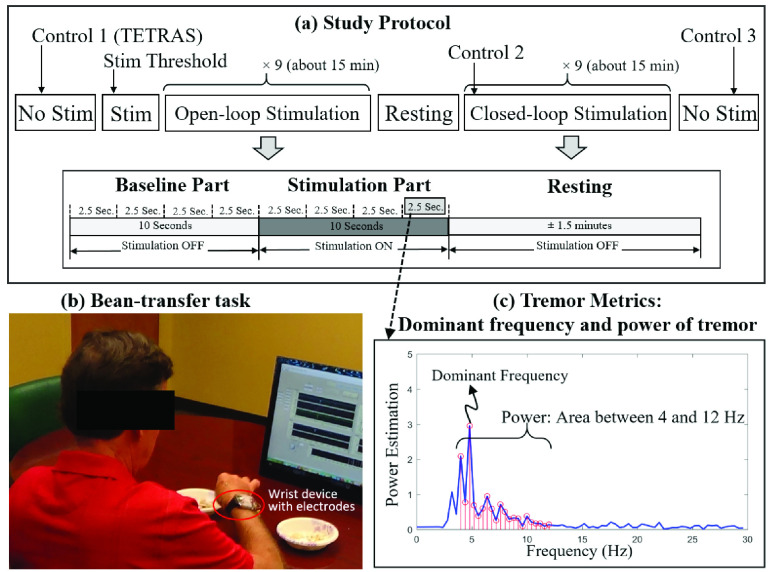
(a) Overall study protocol with a detailed explanation of each stimulation trial that consisted of baseline, stimulation, and resting segments. (b) Actual experimental setup for the bean transfer tasks: One of the subjects performed the bean-transfer task while wearing the wrist device and electrodes. (c) Example of the frequency domain analysis of tremor signals. The power spectral density (PSD) of tremor movement yielded the dominant tremor frequency [29] and the tremor power (integral of power between 4 and 12 Hz) [33]. (d) Modified definition of TETRAS score [29], [30], based on tremor movement amplitude.

The system is comprised of four components: (1) a wireless wrist device that consists of a sensor interface and a constant voltage stimulator (Figs. 2b-e), (2) a wireless transceiver (Fig. 2h), (3) a pair of gel-based surface electrodes (Fig. 2d), and (4) a graphical user interface (Fig. 2g) with a signal-processing algorithm (Fig. 2a). The wrist device incorporates a three-axis motion sensor (LSM303D, STMicroelectronics), a microcontroller (CC2510, Texas Instruments), a wireless transceiver (2.4-GHz radio frequency), a custom-built constant voltage mode stimulator circuitry (Fig. 2b), and a rechargeable 3.7 V lithium-ion battery. The custom-designed electronics (on an 18 $\times$ 28 mm^2^ circuit board) are enclosed in a commercially available wrist band (Fig. 2e). The battery is charged by a standard linear lithium-ion battery charger (LTC4054, Linear Technology) connected to a 5 V mini-USB adapter. A full charge takes about three hours.

The peripheral-nerve stimulation is generated by a voltage-mode stimulator. We chose to use a voltage-mode device (instead of a current-mode device) to avoid the risks of constant-current stimulation modes, such as the induction of skin burns if the resistance between the surface electrode and the skin was abnormally high because of a loss of adhesion [28]. We use a boost converter (LM27313, Texas Instruments) to step up the voltage level of the supply (3.7 V battery) to as high as 20 V for use as stimulation. To generate biphasic stimuli (up to ±20 V, depending on the output of the boost converter), two electronic switches ($\Phi _{1}$ and $\overline {\Phi }_{1}$ in Fig. 2b) are switched on and off alternatingly. The stimuli are applied as short stimulation trains. The amplitude of stimulation is regulated by a variable resistor (AD5162, Analog Device) controlled by the MCU via a serial peripheral interface (SPI). We are also able to vary the duration of each stimulus (set at $200~\mu \text{s}$ for this study), the frequency of stimulation, the number of stimuli per stimulus train (determining the duty cycle), and the onset of stimulation trains (in this study adjusted to coincide with specific phases of the tremor cycle). The stimulation voltage generated by the wrist device is conveyed to the peripheral nerve via a pair of surface electrodes (0.8” round transcutaneous electrical nerve stimulation unit electrodes, Syrtenty).

### B. Tremor Detection Algorithm and User Interface

To identify tremor, we high-pass filtered the quadratic mean of the threeaxis accelerometer data ($\text{A}_{\mathrm {QM-xyz}}$; Eq. (1)) for each sample (every 10 ms) [34] (> 3 Hz).

\begin{equation*} A_{QM-xyz}=\sqrt {\frac {1}{3}(A_{x}^{2}+A_{y}^{2}+A_{z}^{2})}\tag{1}\end{equation*}

The resulting data was analyzed to determine the dominant tremor frequency and peak amplitude of individual tremor movements while the patients performed the bean-transfer task without stimulation (calibration mode). We defined the tremor characteristics during calibration mode as *“baseline tremor”*, and “*active tremor”* during this period as epochs in which tremor reached at least 60% of the maximal amplitude, with a frequency within a ±30% range of the dominant frequency of the baseline tremor. Once the sensor data was streamed in real time, we analyzed the filtered quadratic mean sensor output to identify active tremor periods and tremor phase. For both open- and closed-loop stimulation sessions, the computer sent the signal for stimulus onset to the wrist device via the wireless transceiver (Fig. 3a) when the onset of individual tremor cycles was detected.

The graphical user interface (GUI) was implemented on a personal computer using LabVIEW 2016 (National Instruments). The GUI has three experimental modes: (1) a calibration mode, in which tremor is measured in the absence of stimulation, (2) an open-loop experimental mode, in which constant frequency stimulation trains is applied over the whole stimulation trials, and (3) a closed-loop experimental mode, in which phase-locked stimulation is used to modulate tremor in real time. The GUI also controlled the stimulation parameters, including the amplitude, duty cycle, and frequency of stimulation, and the tremor phase at which stimuli were applied. Detailed specifications of the wearable peripheral-nerve electrical stimulation system are summarized in Table 1.

**TABLE 1:** Wearable Peripheral Nerve Stimulation Specifications

Specification	Value
Control Unit (Wrist Device)	
Microcontroller Unit	CC2510 2.4-GHz RF
Control Unit Dimensions	28 $\times$ 18 mm^2^
Power Source	3.7 V 300 mAh Li-ion battery
Board Weight (with Battery)	5 grams (11 gram)
Total System with Enclosure	36 grams
Sampling Rate	100 Hz
Operating Hours	~ 20 Hours
Accelerometer Sensor Module	
Accelerometer	LSM303D
Sensitivity	0.061 mg/LSB(±2g)
Stimulator	
Stimulation Voltage Range	± 20 V
Channel	1
Adjustable Parameters	Amplitude, frequency, duty cycle, phase
Electrode	
Size	0.8” Round
Material	Silver Tan Tricot Electrodes

### C. Stimulation Parameters

For this experiment, we stimulated branches of the radial nerve (Fig. 3e). One stimulation electrode was placed on the skin over the radial nerve near the wrist, and the other electrode on the skin of the arm about 2 cm apart from the first electrode (see Figs. 2e and 3b). In initial studies in each subject, we determined the minimal strength of stimulation that could be discerned by the subject (“1T”). The thresholds ranged from 3.57 V to 17.33 V (mean: 11.06 ± 5.00 V) with single (biphasic) stimuli (see Table 2). We explored a range of stimulation parameters (i.e., frequency and duty cycle) with open-loop continuous stimulation (Fig. 3a) and phase-locked stimulation (Fig. 3b). The selection of stimulation parameters used in this study are based on those used by other investigators in previous peripheral nerve stimulation studies for tremor suppression [16]–[24] and for stimulation of sensory fibers [28]. We applied nine combinations of stimulation parameters based on three stimulation frequencies (freq. = 50, 100 and 200 Hz; Fig. 3c) and three duty cycles (12.5%, 25% and 37.5% of tremor cycle; Fig. 3d). The following parameters were kept constant: amplitude = 1T (sensory threshold), pulse duration $= 200\,\,\mu \text{s}$ (biphasic; $100~\mu \text{s}$/phase), and phase = 0 (only for the closed-loop phase-locked stimulation session; Fig. 3b) on the radial nerve (Fig. 3e).

**TABLE 2:** Participant Demographics

Participant	Age, years	Sex	Year Since diagnosis	Dominant Hand	Handedness (EHI) Score	Tremor Presence (Dominant Side)	TETRAS Score (L /R)	Medication	Voltage of 1T of Electrical Stimulation (± V)	Wrist Device Placement
ET 01	70	M	50	R	4.00 ± 0.00	Both	2 / 3	N/A	15.39	R
ET 02	76	F	8	R	4.45 ± 0.52	R	0 / 3	Primidone	9.80	R
ET 03	75	M	30	R	4.82 ± 0.40	Both (L)	4 / 4	{Propranolol, Primidone, Zonisamide}^§^	17.33	R^*^
ET 03^**^							3 / 2		11.74	
ET 04	58	F	10	R	5.00 ± 0.00	R	0 / 4	Primidone	5.67	R
ET 05	64	M	15	L	1.00 ± 0.00	Both (L)	4 / 1	N/A	12.96	L
ET 06	58	F	10	R	5.00 ± 0.00	Both (R)	1 / 1	Trihexyphenidyl	4.30	R
ET 07	82	F	10	L	2.18 ± 0.60	Both (L)	2 / 1	Topiramate	15.63	L
ET 08	79	M	10	R	5.00 ± 0.00	Both (L)	4 / 3	N/A	14.17	R^*^
ET 09	47	F	4	R	5.00 ± 0.00	Both (L)	2 / 2	N/A	3.57	R^*^

M: Male, F: Female, R: Right, L: Left, Freq.: Frequency, Amp.: Amplitude;

^*^ indicates that the dominant hand side is not the same as tremor dominant side;

^**^ represents the second session

^§^ indicates the medication applied multiple times in a day.

Bold numbers in $TETRAS~ Scores$ section represent the scores from dominant hand.

### D. Tremor Metrics

As the participants performed the bean-transfer task (explained in Section II. F), we rated the severity of tremor with the Essential Tremor Rating Assessment Scale (TETRAS, see Fig. 4d) [29], [30] during the control trial. We analyzed tremor using two metrics: (1) the *dominant frequency* of the tremor [31], [32], and (2) the *power* of the tremor between 4 and 12 Hz [33]. To analyze these tremor metrics, we first applied a tenth-order Butterworth high-pass filter (>3Hz) to the series of quadratic mean values of the three-axis accelerometer (Eq. (1)). We then applied fast-Fourier transforms (FFTs) for spectral analyses (sampling frequency (Fs) = 100 Hz, sampling period = 2.5 s, length of signal = 250 samples).

We defined the *dominant tremor frequency* as the peak frequency of the power spectral density (PSD; Fig. 4c) and the *tremor power* as the integral of the PSD output between 4 and 12 Hz (Fig. 4c) Eq. (2):

\begin{align*} \text {Power}=&\int _{f_{a}}^{f_{b}}\frac {FFT^{*}(tremor)\times FFT(tremor)}{N^{2}}df \\ f_{a}=&4 Hz, f_{b}=12~Hz;~\hbox {[{33}]}\tag{2}\end{align*}

The * denotes the complex conjugate, N represents the number of sample points, and g^2^/Hz (g is equal to 9.8 m/s^2^) is the unit of the tremor power.

To assess the relative changes in the tremor output metrics, we defined each normalized metric as a ratio of the output metric of the baseline segment to that of the stimulation segment of each trial in Eq. (3):

\begin{equation*} \textit {Normalized metric} =\frac {\textit {Metric from stimulation part}}{\textit {Metric of average baseline part}}\tag{3}\end{equation*}

### E. Subjects

This study was approved by the Institutional Review Board (IRB) of the Georgia Institute of Technology. To evaluate the performance of the wearable tremor modulation system, we conducted experiments in nine ET participants, one of whom participated in two sessions on different days. We recruited participants with diagnoses of ET and with kinetic tremor in at least one arm. The ET participants were recruited from the Atlanta and Savannah, GA area. The participants were diagnosed by their respective neurologists. The participants remained on their regular medication. Information about these participants is summarized in Table 2. Each participant signed a written informed consent prior to the experiment. We collected tremor data with and without peripheral-nerve electrical stimulation from our wearable tremor modulation device.

We used the Edinburgh Handedness Inventory (EHI) questionnaire [35] to determine the handedness of our subjects (Table 2). Participants wore the wrist device on their dominant hand: seven on their right and two on their left. Although three of the right-handed subjects had left-side dominant tremor, they also experienced tremor on the right side. The subjects reported the side on which they experienced the worse tremor, summarized in Table 2.

### F. Experimental Design and Procedure

The participants completed a questionnaire regarding their tremor history, the presence of their tremor side(s), and their dominant hand. The information from the questionnaire is summarized in Table 2. To evaluate the effect of stimulation on tremor movement, the subjects performed the bean-transfer task, which involves use of a spoon to transfer a medium-sized lima bean from one plate to another (see Fig. 4b). The subjects were required to use wrist movements to pick up one bean from the plate, and move it to another plate, 30 cm away from the first. We first collected data for ten seconds (control data) in the absence of stimulation (Fig. 4a) to analyze the dominant frequency and power, (shown in Table 3 and Figs. 6a-b). These data for each subject were used for the tremor detection algorithm.

**TABLE 3:** Summary of Tremor Movement Measurements for the Control Trial

PT #	Tremor Frequency (Hz)	Tremor Power (g^2^/Hz)
ET01	6.00 ± 0.33	8.44 ± 0.93
ET02	5.60 ± 0.01	9.10 ± 3.23
ET03	4.80 ± 0.33	13.65 ± 2.34
ET04	5.90 ± 0.50	12.80 ± 1.34
ET05	4.93 ± 0.23	23.46 ± 1.73
ET06	6.80 ± 1.31	4.97 ± 1.20
ET07	5.10 ± 0.38	4.47 ± 1.12
ET08	6.50 ± 0.38	10.61 ± 1.71
ET09	6.70 ± 0.95	7.75 ± 1.98
ET03^**^	4.80 ± 0.65	7.43 ± 1.74

^**^ Represents the second session.

Stimuli were applied using the stimulation parameters mentioned above. To determine the stimulus amplitude, we placed a pair of electrodes over a branch of the radial nerve on the wrist (Figs. 2d and 4b) and adjusted the placement of the electrodes so that the subjects sensed the stimulus in the desired location (see Fig. 3e, shaded area). This process took less than two minutes, after which no subsequent stimulation was applied for five minutes to minimize residual effects.

Next we performed two stimulation sessions: applying nine combinations of stimulation parameter sets in (1) continuous, constant-frequency (dominant tremor frequency) open-loop stimulation (Fig. 3a) and (2) phase-locked closed-loop stimulation (Fig. 3b). Each session consisted of nine stimulation trials, using the different parameter combinations mentioned above, and each consisting of a ten-second baseline (no stimulation) segment, a ten-second stimulation segment, and ±1.5 minutes of rest. During the baseline and stimulation segments of each trial, the subjects were asked to perform the bean-transfer task. We analyzed the ten-second baseline/stimulation period in four 2.5-second segments each. Although we did not control the speed of all arm movements, most participants transferred four beans during the stimulus period. During each trial, we randomly selected one of the nine combinations of stimulation parameter sets and asked the subjects to perform the open-loop stimulation session first and then the closed-loop stimulation session. Between sessions, the subjects had an extra rest period. Each stimulation session took about 15 minutes (for a total of 30 minutes of study).

Following the stimulation sessions, the participants responded to a five-point Likert-scale questionnaire consisting of seven questions about the system, the stimulation, and their tremor condition. The seven questions of the questionnaire are summarized in Table 4, and the responses of the nine participants are summarized in Fig. 8.

**TABLE 4:** Qualitative Assessment for the Wearable Tremor Modulation System

Questionnaire after the experiment:	
Q1.	Comfort of the wearable tremor modulation system: 1-Very uncomfortable, 3-Normal, 5-Very comfortable
Q2.	Tremor during stimulation compared to the normal condition: 1-More severe, 3- the same as the normal condition., 5- Less severe
Q3.	Comfort of electrical stimulation: 1-Very uncomfortable, 3-Normal, 5-Very comfortable
Q4.	Pain from electrical stimulation: 1-Very painful, 3-Normal, 5-Not painful at all
Q5.	Tingling from electrical stimulation: 1-A large amount of tingling, 3-Normal, 5-Not tingling at all
Q6.	Strength of electrical stimulation: 1-Very strong, 3-Normal, 5-Not strong at all
Q7.	Willingness to use this for potential treatment (overall satisfactory): 1-Very negative, 3-Normal, 5-Very positive

**FIGURE 5.**
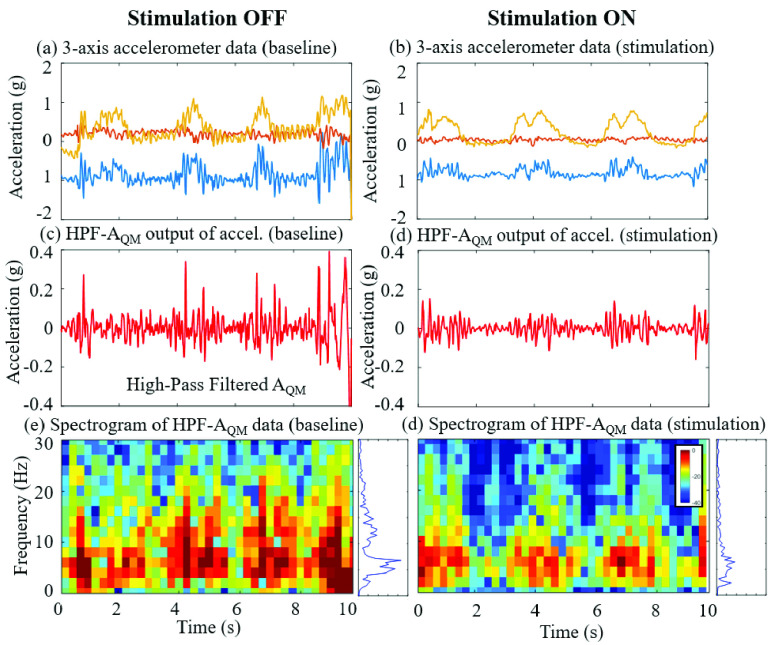
Tremor recording obtained during a control trial (stimulation OFF, a, c, and e) and a stimulation trial (stimulation ON, b, d, and f) for participant ET04. Raw three-axis accelerometry data obtained while the patient performed the bean-transfer task are shown in (a) and (b), while (c) and (d) show results of the HPF-$\text{A}_{\mathrm {QM}}$ output of the same data. The plots in (e) and (f) show spectrograms (window: 1/2 sampling frequency (Fs), overlap: Fs) and the average power density for the data shown in (c) and (d).

**FIGURE 6.**
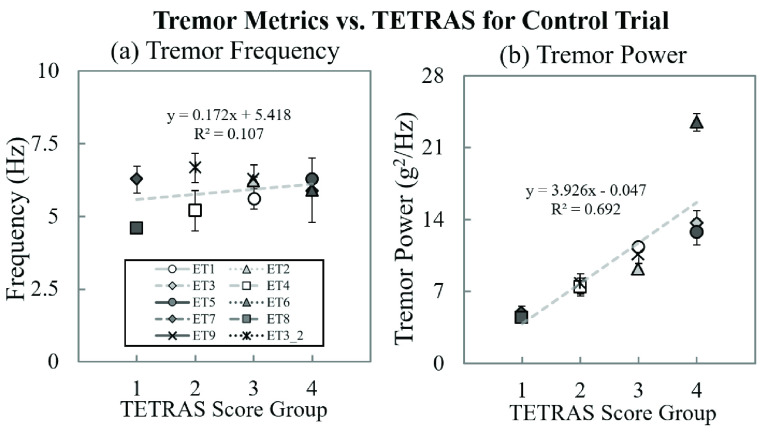
Summary of the tremor movements (control trials) that were quantitatively analyzed with two tremor metrics: (a) dominant tremor frequency, and (b) tremor power compare the metrics and TETRAS scores. The error bar represents the standard error.

**FIGURE 7.**
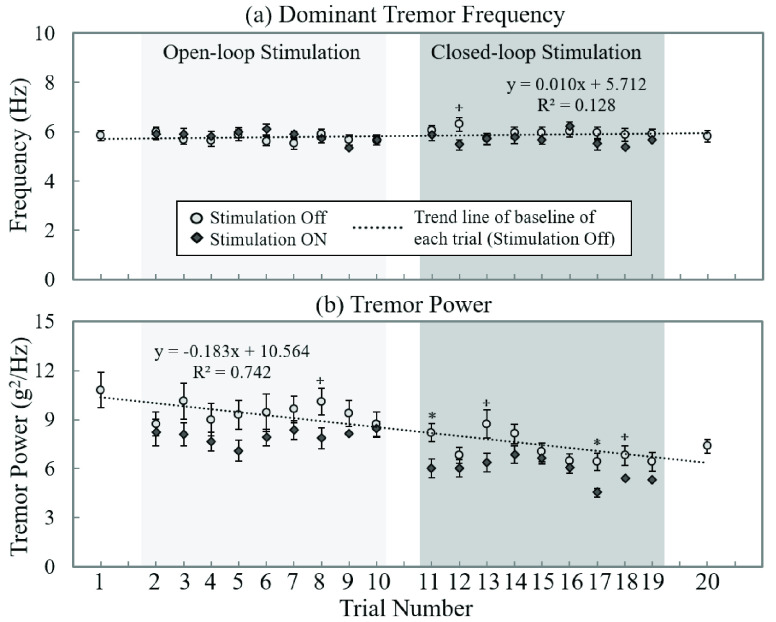
Effects of peripheral nerve stimulation over the course of trials. Overall tremor movement changes without and with stimulation for (a) dominant tremor frequency and (b) tremor power. The error bar represents the standard error; $^\star $: P < 0.05; *: P < 0.005.

**FIGURE 8.**
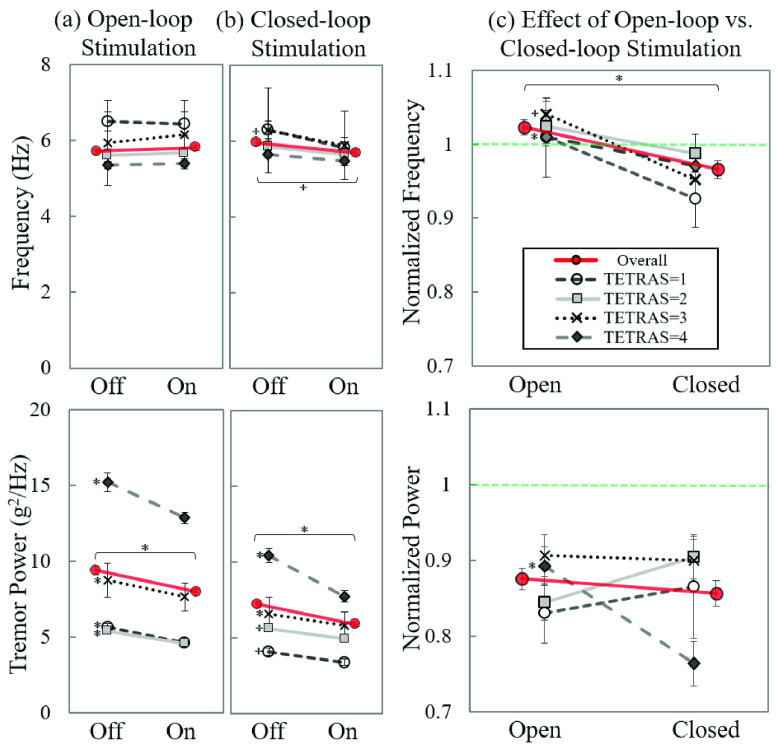
Comparison of open- vs. closed-loop stimulations by TETRAS score groups. Average tremor metric changes by the (a) open-loop and (b) closed-loop stimulations of all nine combinations of stimulation parameter sets. (c) Effect of open- vs. closed-loop stimulations in normalized metrics. Graphs in upper panel represent dominate tremor frequency, and those in lower panel represent tremor power. The error bar represents the standard error; $^{\star }$: P < 0.05; *: P < 0.005.

### G. Statistical Analysis

We conducted t-tests ($\alpha =0.05$ and 0.005) to determine the differences between epochs with and without stimulation for individual subjects and across all subjects. We also conducted a pairwise linear regression analysis to determine the correlation between tremor metrics (frequency, power, and TETRAS score).

## III. Results

We analyzed the severity of tremor both without stimulation and with stimulation, using both quantitative measurements and TETRAS scores. Figs. 5a-f shows an example dataset for a single participant (ET04) with a TETRAS score of 4. The stimulation reduced tremor in this participant. The analyses show that the stimulation reduces the tremor for this participant.

### A. Movements During the Control Trials

Table 3 shows results from all subjects obtained while they performed the bean-transfer task without stimulation. The overall dominant tremor frequencies were between 4.40 and 9.20 Hz (mean ± SE: 5.83 ± 0.18 Hz) and the tremor power was between 3.60 and 24.94 g^2^/Hz (mean ± SE: 10.80 ± 0.96 g^2^/Hz).

Figs. 6a-b show the correlation between each tremor output metric and TETRAS scores [34]. We found a high correlation (using a linear regression analysis) between tremor power and TETRAS scores in ten experimental sessions (R^2^ = 0.692). The dominant frequency was independent of TETRAS score (R^2^ = 0.107).

### B. Tremor Fluctuation Over Time

To evaluate the effect of peripheral nerve stimulation over time, we analyzed tremor fluctuation with and without stimulation during the bean-transfer task during all trials. The average results of all patients are plotted in Figs. 7a-b. Trials 1 and 20 were control trials; trials 2–10 and trials 11–19 were open- and closed-loop stimulation trials, respectively. While the dominant tremor frequency did not change significantly over the course of the trials (R^2^ = 0. 128; Fig.7a), tremor power decreased linearly (R^2^ = 0.742; Fig.7b). In most trials, the stimulation decreased tremor power compared to its baseline (not all trials showed statistically significant decreases). The study protocol included a sufficient rest period (about 1.5 minutes; at least ten times as long as the stimulation period) between the trials; we observed, however, that the effects of stimulation on tremor power may have been lasting, but gradually reduced the baseline tremor power during the entire course of the trials (about 30 minutes). To evaluate the stimulation effect for all 18 trials, we also analyzed and compared the normalized tremor power and the tremor power from the control trial (trial 1). We estimated the overall rate of tremor power reduction at 42.17 ± 3.09% (TETRAS=1: 32.67 ± 13.90%, TETRAS=2: 27.59 ± 3.58%, TETRAS=3: 44.14 ± 3.69%, and TETRAS=4: 60.05 ± 5.29%) at the last stimulation trial (trial 19) that compared with the control trial (trial 1); we observed the greatest reduction in tremor power during trial 17 (closed-loop stimulation) from the most severe tremor groups (TETRAS=4) as 70.70 ± 6.30 %.

### C. Effect of Open-Loop vs. Closed-Loop Stimulation

The dominant tremor frequency by open-loop stimulation was unchanged (Off: 5.71 ± 0.07 Hz and On: 5.81 ± 0.07 Hz, P = 0.340; Fig. 8a-up), while closed-loop stimulation resulted in a significant reduction (Off: 5.96 ± 0.08 Hz and On: 5.68 ± 0.07 Hz, P = 0.010; Fig. 8b-up). The tremor power significantly decreased by both open- (Off: 9.37 ± 0.30 g^2^/Hz and On: 7.97 ± 0.23 g^2^/Hz, P < 0.001; Fig. 8a-down) and closed-loop stimulation (Off: 7.22 ± 0.20 g^2^/Hz and On: 5.91 ± 0.16 g^2^/Hz, P < 0.001; Fig. 8b-down).

When we analyzed the effects of open- and closed-loop stimulation with the normalized tremor metrics (Eq. (3)), we found that the average normalized dominant tremor frequencies of the open- and closed-loop stimulation differed significantly (open: 1.02 ± 0.01, and closed: 0.97 ± 0.01; P < 0.001, Fig. 8c-up). The normalized tremor power was analyzed as 0.88 ± 0.01 and 0.86 ± 0.02 for open- and closed-loop stimulations, respectively, which indicates that both forms of stimulation reduced tremor power by about 12–15% compared to the corresponding baseline period, with no difference between open- and closed-loop stimulation sessions (Fig. 8c-down; P = 0.373). The normalized frequency declined across all TETRAS score groups, while the normalized power remained unchanged.

We conclude that the dominant tremor frequency can be modulated by the phase-locked closed-loop stimulation, whereas tremor power was similarly reduced by open- and closed-loop stimulations. Since the open-loop stimulation was continuous while the closed-loop stimulation was only delivered when tremor was detected (50–70% of each trial), we conclude that phase-locked closed-loop stimulation is a more efficient mode of tremor reduction.

### D. Effect of the Frequency and Duty Cycle of Stimulation

**FIGURE 9.**
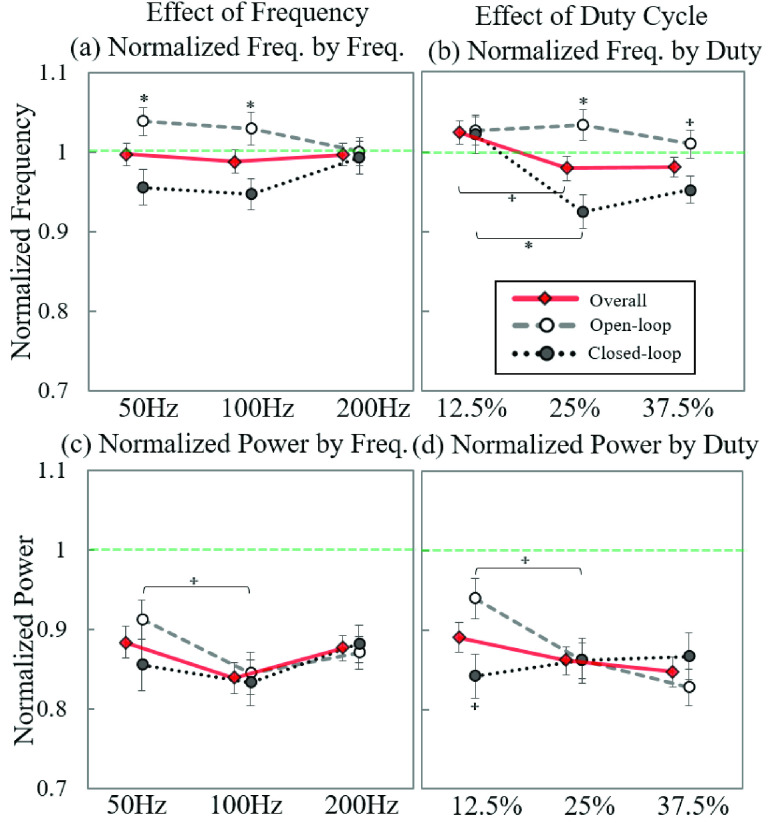
Effects of frequency and duty cycle of stimulation in normalized metrics: (a) the normalized dominant tremor frequency by stimulation frequencies, (b) the normalized dominant tremor frequency by stimulation duty cycles, (c) the normalized tremor power by stimulation frequency, and (d) the normalized tremor power by stimulation duty cycles. The error bar represents the standard error; $^{\star }$: P < 0.05; *: P < 0.005.

We analyzed the effects of the stimulation frequency and the duty cycle with the normalized tremor metrics by open- and closed-loop stimulation separately (Figs. 9a-d). The overall ratio of the dominant tremor frequency was not affected by the stimulation frequency when results of open- and closed-loop stimulation trials were combined. When we separately analyzed the effects of stimulation frequency by open- and closed-loop stimulations, we found that the open-loop stimulations increased the dominant tremor frequency at the 50 and 100 Hz stimulation frequencies but closed-loop stimulation decreased frequencies (Fig. 9a). As expected, the ratio of the dominant tremor frequency of the 50 and 100 Hz stimulation frequencies differed significantly between open- and closed-loop stimulations.

The stimulation frequency of 100 Hz showed the lowest ratio of tremor power for both open- and closed-loop stimulations, but they did not statistically differ (except for the open-loop stimulation of 50 and 100 Hz stimulation frequencies; Fig. 9c).

The overall ratio of the dominant tremor frequency also remained in a similar range during the three stimulation duty cycles (Fig. 9b); the overall ratio of the dominant tremor frequency slightly increased by 12.5% (a significant difference between 12.5% and 25%), but decreased by 25% and 37.5% (no significant difference between 25% and 37.5%). We found that the dominant tremor frequencies slightly increased during all three-duty cycles for open-loop stimulation but significantly decreased by 25% and 37.5% for closed-loop stimulation.

The ratio of tremor power also remained similar across the different stimulation duty cycles, with a slight tendency toward a higher tremor reduction during the longer duty cycles. The only statistically significant changes were found at a 12.5% duty cycle between open- and closed-loop stimulations; at this level of stimulation, the ratio of tremor power was least affected by open-loop stimulation but most strongly affected by closed-loop stimulation.

We conclude that the 50 and 100 Hz stimulation frequency and 25% and 37.5% duty cycles for closed-loop stimulation significantly reduced the tremor frequency, and the 100 Hz stimulation frequency and 37.5% of the duty cycle for open-loop stimulation was most effective at reducing tremor power.

### E. Qualitative Assessment

**FIGURE 10.**
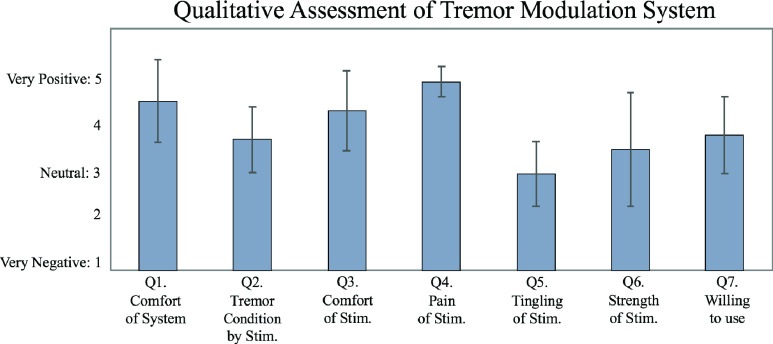
Summarized results of the qualitative assessment of nine ET participants after the experimental trials. Error bar represents the standard deviation.

After the experiment, the participants responded to a qualitative assessment questionnaire based on a five-point Likert scale consisting of seven questions (Table 4). The questions assessed whether the system was comfortable to use, fatigue from the experiment, whether participants sensed the electrical stimulation itself, and whether they thought that the stimulation affected their tremor. The averaged values of the responses of the nine subjects (one of whom participated in two sessions; total n=10) are summarized in Fig. 10. The responses indicate that both the system and stimulation were comfortable (Q1 (mean ± SD): 4.50 ± 0.85 and Q3: 4.30 ± 0.82, respectively). They also indicated that none of participants experienced pain from stimulation (Q4: 4.90 ± 0.32), yet they felt some tingling and were aware of the strength of the stimulation (Q5: 3.00 ± 0.67, and Q6: 3.50 ± 1.18, respectively). Six of the ten responded that their tremor was less severe (answered as 5 of Likert scale) during the stimulation trials than in their normal condition (Q2: 3.70 ± 0.67), and six of them felt positive or very positive about the potential use of the wearable tremor modulation system for their potential treatment of tremor (Q7: 3.80 ± 0.79).

## IV. Discussion

We designed and implemented a wearable device that allows us to stimulate peripheral nerves with different combinations of stimulation parameter sets to tremor movements. Initial testing of the device demonstrated that the stimulation is well tolerated by patients with ET and that it can lead to measurable improvements in ET severity. The effects were relatively small, but it should be emphasized that our patients were fully treated with medications at the time of testing, so that the present results would reflect effects that are synergistic between medication and stimulation.

### A. Comparison to Previous Studies

Previous attempts at modulating tremor characteristics were conducted with a range of (empirically determined) stimulation parameters [16]–[24], [28]. These studies applied biphasic pulses, generally at frequencies between 30 and 150 Hz, with pulse durations of 100 and $400~\mu \text{s}$ per phase (the pulse duration in one study was as high as $1000~\mu \text{s}$ [28]). The duration and/or the phase of stimulation was typically 50% of the full tremor cycle out-of-phase. We used stimulation conditions that were within the range of these published parameters, with the addition of phase-locking of the stimulation to the real-time tremor cycle. To maximize the effectiveness of stimulation from the wearable device, we need to understand the roles of stimulation parameters for real-time parameter optimization. Therefore, we plan to further analyze the effects of a larger range of stimulation parameters on the tremor metrics in the future.

Most of the other studies use constant-current stimulation by either surface or needle electrodes. We developed our wearable wrist device with a constant-voltage design instead, using off-the-shelf components to reduce the size of the device, and to minimize the possibility of unexpected high current flow to the surface electrodes. One study [28] showed that the voltage-mode stimulator did not deliver a sensation that differed from that of the current-mode stimulator via surface electrodes, presumably because the impedance of the electrode, the electrode-skin interface, and the underlying tissue remains relatively constant during the stimulation. Therefore, we expect that the results of constant-voltage stimulation would be similar to those of constant-current stimulation.

As mentioned above, external electrical stimulation for the control of tremor has been examined in patients with parkinsonian tremor or ET, using non-wearable devices, and stimulation strategies that included out-of-phase muscle stimulation [16]–[18], [21], [24], or cutaneous stimulation [19], [20], [22], [23]. These studies [16]–[25] used stimulation of either muscles or branches of one or several of the major nerves supplying the arm (median, ulnar and radial nerve). In the present study, the stimulation was applied to branches of the radial nerve. Exploration of the effects of stimulation of median or ulnar nerves would obviously also be interesting (and feasible).

We compared the effects of various types and levels of stimulation on tremor movements, listed in Table 5. Although muscle stimulation from [17], [18], [24], [25] reported a promising tremor reduction ratio of between 50% and 85%, the threshold amplitude of the stimulation to generate muscle twitches are typically higher than the sensory threshold. Sensory stimulation with various stimulation mechanisms [19]–[24] also showed a tremor reduction ratio of between 14 and 60%, which is somewhat less effective than that with muscle stimulation. Particularly, one study [23] also developed/evaluated a wearable stimulation device on the wrist to modulate tremor. The authors applied stimulation continuously for 40 minutes to compare the severity of tremor before/after stimulation (14–22% of TETRAS score reduction). Even though our study implemented different stimulation approaches (i.e., constant voltage mode, stimulation parameters), our peripheral nerve electrical stimulation study using the fully wearable closed-loop system showed a reduction in tremor of about 41% (up to 70%), and the phase-locked stimulation showed some potential for a reduction in tremor and a modulation of its frequency.

**TABLE 5:** Quantitative Comparison of Tremor Suppression Using Electrical Stimulation

Reference	Stimulation Location	Patients	Stimulation Duration	Strategy	Efficacy
Prochazka et al., 1992 [17]	Wrist (flexor and extensor)	3 ET, 4 PD, 6 other	20 min	Functional electrical stimulation (FES), closed-loop out-of-phase stimulation	ET: 73%; PD: 62%; Others: 38%
Gillard et al., 1999 [18]	Wrist/Finger (flexor and extensor)	3 PD	30s per trial, 10 trials	Muscle closed-loop out-of-phase stimulation	84.5 ± 2.2%
Hao et al., 2013 [19]^*^	Hand (cutaneous afferents)	11 PD	40s per trial (10s no stim-20s stim-10s no stim), 10 trials	Artificial cutaneous afferents	N/A
Xu et al., 2016 [20] ^*^	Finger (radial nerve)	2 PD	15s per trial (5s no stim-5s stim-5s no stim), 10 trials	Closed-loop transcutaneous electrical nerve stimulation (desktop device)	About 50% reduction (joint angles)
Dideriksen et al., 2017 [21] ^*^	Wrist/Finger (flexor and extensor)	5 PD, 4 ET	150s per trial (stim of/off 30s windows),10 trials	Below the motor threshold, out-of-phase pattern; surface electrodes vs. intermuscular electrodes	Average highest suppression: 52% (54% intermuscular, 50% surface)
Lin et al., 2018 [22] ^*^	Wrist (median and radial nerves)	23 ET	40 min	Peripheral nerve stimulation (desktop device)	TETRAS: 60 ± 8.4%
Pahwa et al., 2019 [23] ^*^	Wrist (median and radial nerves)	77 ET (N=40 for stim)	40 min	Peripheral nerve stimulation (wearable device)	Spiral rating: N.S, TETRAS: 14–22% reduction, ADLs (subjective rating): 49%
Dosen et al., 2014 [24] ^*^	Wrist (flexor and extensor)	2 ET, 4 PD	2 min per trial, 5 trials for each mode	Out-of-phase pattern; Sensory (< thrm) vs. motor (>thrm) stim.	60 ± 14% (> thrm), 42 ± 5% (< thrm)
Gallego et al., 2013 [25]	Wrist (flexor and extensor)	4 ET, 2 PD	30s per trial 6–12 trials	Muscle co-contraction at a pair of antagonists	52.33 ± 25.48%
This study^*^	Wrist (Radial nerve)	9 ET (N=10)	20s per trial (10s no stim-10s stim) 18 trials	Sensory stimulation (wearable device) via peripheral nerve stimulation; open vs. closed-loop stimulation	42.17 ± 3.09 % (up to 70.70 ± 6.30%)

N/A: Not available, N.S: not significant, ADLs: activities of daily living,

^*^ : study utilized with sensory or sub-threshold stimulation.

### B. Study Limitations

All participants of this study experienced tremor during movements of their arms, with a range of amplitudes that varied depending on the task being performed. We administered a specific task, the bean-transfer task, that mimicked utensil use while eating (an activity frequently disrupted by tremor). This task requires only a limited number of joint movements. In future studies, tasks requiring a richer repertoire of movements and postures will have to be tested.

A potential confound is that we did not control the medications of the subjects. Instead, patients took their usual tremor treatment. As shown in table II, 5 of our participants took a typical anti-tremor medication. However, as our trial is a trial of a potential symptomatic treatment for tremor, it is important to emphasize that all participants showed tremor during the testing session. Control and stimulation sessions for each participant were run sequentially, so that it can be assumed that medication effects (if any) applied to both equally. More rigorous future testing needs to involve dedicated tests on and off their medications.

Another limitation of this study is that the close-loop stimulation session always took place after the open-loop session. Even though we allowed a sufficient rest period between stimulation trials and between open- and closed-loop stimulation sessions, we still saw a linear decrease over the course of the trials. We attempted to compare the effect of each stimulation trial with its baseline tremor characteristics but were unable to avoid the residual effects from the previous stimulation.

The HPF-$\text{A}_{\mathrm {QM}}$ data generated from the accelerometer output did not indicate the origin of the tremor movements (i.e., joint/muscles). To gain a more detailed understanding of the origin of tremor and the effects of stimulation, electromyographic (EMG) analyses of the activity of muscles of the tremulous limb could be conducted. However, the fully wearable system would in all likelihood not benefit from the inclusion of complex EMG recordings because the design of this system would need to emphasize robustness and low energy consumption.

Finally, the study personnel conducting TETRAS scoring was not clinically trained or blinded. Although we carefully followed the definition of TETRAS scores in Fig. 3e [29], [30], it would have been better to rely on neurologically trained personnel to score the patients, and to conduct the entire study in a blinded manner to avoid biases in the analysis. We wish to remind readers, however, that the current study was a proof-of-principle experiment, exploring the potential use of the external stimulation device. Future studies will benefit from a larger number of subjects and a more robust study design that includes blinded, medically trained raters.

### C. Potential of This Study

In this study, we tested a wearable peripheral nerve electrical stimulation system with real-time parameter updates to modulate tremor movements. Further improvements will include the use of more advanced control algorithms that allow fully functional real-time optimization of stimulation parameters across a larger parameter space and the implementation of an off-board signal processing function/optimization algorithm on a smartphone via Bluetooth connectivity for realistic mobile usability.

The development of a wearable tremor modulation system based on the electrical stimulation of peripheral nerves is expected to have a substantial clinical impact. If studies of larger patient populations confirm the efficacy of this stimulation method, this technique will offer a simple non-invasive and cost-efficient option for alleviating the symptoms of patients with medication treatment-resistant kinetic tremor. The method may be particularly effective in patients who do not qualify for conventional surgical approaches to tremor treatment such as DBS. Such patients include those with medical or surgical contraindications to surgery (e.g., the presence of significant medical or psychiatric disease), those unwilling to undergo invasive brain surgery, and those with inadequate resources or access to qualified post-operative care. The proposed non-invasive tremor treatment system could also be combined with other available approaches (e.g., medications, surgery) to optimize the efficacy of a patient’s overall treatment. As the successful completion of future validation studies could expand the spectrum of practical neuromodulation techniques, larger segments of the patient population could have access to them.

## V. Conclusion

The purpose of this study was to propose a real-time wearable tremor modulation system that provides constant-voltage peripheral-nerve stimulation and to quantitatively assess the feasibility of such stimulation in individuals with ET. To evaluate the effects of peripheral-nerve stimulation delivered by a wearable wrist device, we collected tremor data from patients with ET, with and without stimulation, and analyzed stimulation-related changes in their movements using the dominant tremor frequency, tremor power, and the frequency deviation as evaluation metrics. The results showed that our system significantly reduced tremor frequency and power, demonstrating the potential usefulness of peripheral-nerve stimulation as a treatment for ET.
